# Engineering and Production of the Light-Driven Proton Pump Bacteriorhodopsin in 2D Crystals for Basic Research and Applied Technologies

**DOI:** 10.3390/mps3030051

**Published:** 2020-07-22

**Authors:** Mirko Stauffer, Stephan Hirschi, Zöhre Ucurum, Daniel Harder, Ramona Schlesinger, Dimitrios Fotiadis

**Affiliations:** 1 Institute of Biochemistry and Molecular Medicine, and Swiss National Centre of Competence in Research (NCCR) TransCure, University of Bern, 3012 Bern, Switzerland; mirko.stauffer@ibmm.unibe.ch (M.S.); stephan.hirschi@ibmm.unibe.ch (S.H.); zoehre.ucurum@ibmm.unibe.ch (Z.U.); daniel.harder@ibmm.unibe.ch (D.H.); 2 Department of Physics, Genetic Biophysics, Freie Universität Berlin, 14195 Berlin, Germany

**Keywords:** bacteriorhodopsin, bioelectronics, biomaterial, bionanotechnology, *Halobacterium salinarum*, light-driven proton pump, protein engineering, purple membranes

## Abstract

The light-driven proton pump bacteriorhodopsin (BR) from the extreme halophilic archaeon *Halobacterium salinarum* is a retinal-binding protein, which forms highly ordered and thermally stable 2D crystals in native membranes (termed purple membranes). BR and purple membranes (PMs) have been and are still being intensively studied by numerous researchers from different scientific disciplines. Furthermore, PMs are being successfully used in new, emerging technologies such as bioelectronics and bionanotechnology. Most published studies used the wild-type form of BR, because of the intrinsic difficulty to produce genetically modified versions in purple membranes homologously. However, modification and engineering is crucial for studies in basic research and, in particular, to tailor BR for specific applications in applied sciences. We present an extensive and detailed protocol ranging from the genetic modification and cultivation of *H. salinarum* to the isolation, and biochemical, biophysical and functional characterization of BR and purple membranes. Pitfalls and problems of the homologous expression of BR versions in *H. salinarum* are discussed and possible solutions presented. The protocol is intended to facilitate the access to genetically modified BR versions for researchers of different scientific disciplines, thus increasing the application of this versatile biomaterial.

## 1. Introduction

Since its discovery in 1971 [1], the light-driven proton pump bacteriorhodopsin (BR) from the extreme halophilic archaeon *Halobacterium salinarum* (previously known as *Halobacterium halobium*) has become an intensively studied model membrane protein for proton translocation across biological membranes [2,3,4,5], photochemical events in proteins [6,7,8,9], photophosphorylation [10,11,12] and structural studies [13,14,15,16]. The use of BR to study proton transport across membranes in different time scales using time-resolved serial femtosecond crystallography at X-ray free electron lasers [17,18,19,20] and as an alignment tool for NMR studies [21] highlights its significant contribution to emerging technologies. BR natively occurs in patches in the membrane of *H. salinarum*, called purple membranes (PMs). These patches contain BR and lipids in a hexagonal crystal lattice, with BR forming trimers. This quaternary structure and higher order is responsible for the high thermal stability of PMs [22,23]. Interesting chemical and physical properties, i.e., photochromism, photoelectrism and thermal stability make BR/PMs one of the most promising biomaterials for versatile technical and scientific applications [24].

Upon illumination with light at 568 nm, the covalently bound retinal chromophore of BR undergoes isomerization by absorbing a photon. This induces a photocycle, involving a series of conformational changes and protonation states through different, consecutive states. These spectrally distinguishable intermediates are called K, L, M and O, the last of which thermally decays back to the resting state (bR) [8]. However, if the O intermediate absorbs a second photon (λ_max_ = 640 nm), an alternative intermediate, the thermally stable Q-state, can form enabling long-term storage of information. This feature is exploited in the application of BR in holographic associative processors [25] and volumetric optical memories [26,27]. During a complete photocycle, a proton is transported from the intra- to the extracellular side of the protein, establishing an electric current (photocurrent). This photoelectric effect is applied in photovoltaic devices [28], optical switches [29], optical logic gates [30], light sensors [31], single electron [31]- and field effect transistors [32], chemical sensors [33,34], optical microcavities [35] and may even lead to protein-based retinal implants [36].

The wild-type form of BR or purple membranes is not optimal for numerous applications; thus, mutated forms were engineered to meet specific requirements. Furthermore, variants were needed to conduct studies on mechanistic and structural aspects of BR. For example, in BR-D96N, the duration of the photocycle increases from ~10 ms to ~10 s, which allowed for studying the conformational changes by high-speed atomic force microscopy [37]. The introduction of common protein tags such as the His-tag [38,39] or the creation of fusion proteins such as BR-GFP [40] enable the use of specific biophysical and bionanotechnological techniques. By the modulation of the energetic state of certain photocycle species, BR can be tailored to specific applications. The previously mentioned BR-D96N shows a prolonged lifetime of the M state, leading to an enhanced light sensitivity, which finds application in optical and holographic recordings [28]. In contrast, BR-D85N [41] and BR-V49A/I119T/T121S/A126T [24] show the prolonged lifetime of the O-state, leading to a more efficient formation of the Q-state, which is needed for long-term information storage. BR can also be optimized for photovoltaic applications by enhancing the dipole moment of the purple membranes [24]. If BR is used in photodetector or photovoltaic devices, a high photocurrent is required for efficient operation. A possibility to enhance the photocurrent is increasing the molecular surface density of BR on the used electrode. This was accomplished by engineering the BR-M163C version, which is able to bind to the gold electrode surface via the introduced cysteine residue [42].

The genetic modification of *H. salinarum* was first established using a polyethyleneglycol (PEG)-mediated transformation method [43,44]. The introduction of resistance genes for the antibiotics mevinolin [45,46,47] and novobiocin [48,49] into haloarchaeal plasmids for the selection of transformants and the development of shuttle-vectors [50,51,52], which enabled mutagenesis and vector amplification in *Escherichia coli*, further simplified the transformation of *H. salinarum*. The transformation of purple membrane-deficient strains (e.g., *H. salinarum* L33 [53,54]), in which the bacterioopsin (*bop*) gene is disrupted by transposable elements [54], with plasmids containing wild-type or mutant versions of the *bop* gene, allowed for the homologous expression of purple membranes containing wild-type or mutant versions of BR [50,51]. In parallel, methods for heterologous expression of BR in *E. coli* [55,56,57,58] were developed. However, this approach requires supplementation of retinal to culture media, host-specific signal sequences for membrane targeting, which have to be proteolytically removed later, as well as the purification of BR using detergents followed by reconstitution into lipids to yield functional BR. Furthermore, it was demonstrated that functional BR can be expressed in the yeast *Schizosaccharomyces pombe*, without the need for added signal sequences [59,60]. Importantly, it is not possible to produce BR as purple membranes using these expression systems [55]. It is therefore not recommended to use heterologous expression for applications, which require BR organized in a 2D lattice as found only in purple membranes from halobacteria. Methods for the cultivation of *H. salinarum* [61,62] and the isolation of purple membranes [63] were established early in BR research and were adapted in many scientific publications, including this protocol.

Here, we present a detailed and straightforward description for the production of specifically engineered PMs, from the genetic modification and cultivation of *H. salinarum* to the isolation of pure PMs. Furthermore, we show functional and biophysical techniques for the analysis of those PMs. These methods allow for the production of a wide range of PMs with different forms of mutations, including the addition of different detection and purification tags, the introduction of functional point mutations and the production of fusion proteins. Even chimeras of BR and other proteins were shown to be functionally active, while maintaining the structural integrity of the two-dimensional crystalline PM lattice [64]. Intrinsic problems of the *H. salinarum* expression system are discussed and possible solutions presented.

## 2. Experimental Design

The time needed to perform the experiments in this protocol can strongly vary, depending on the number of iteration cycles needed to achieve a satisfactory clone.

3.1 Site-directed mutagenesis of *bop* in pUC18 (0.5 Day)

3.2 Insertion of *bop-C-His_10_* into pHS blue (0.5 Day)

3.3 Amplification of pHS blue-*bop-C-His_10_* in *E. coli* DH5α (1 Week)

3.4 Preparation of *H. salinarum* L33 cells for transformation (2–3 Days)

3.5 Preparation of plasmid for transformation (10 Min)

3.6 Transformation of *H. salinarum* L33 (2 H)

3.7 Outgrowth of transformed cells (2 Days)

3.8 Plating and colony formation (1–2 Weeks)

3.9 Colony picking and cultivation (1 Week)

3.10 Glycerol stock preparation (30 Min)

3.11 Clone screening by small-volume purple membrane preparation (0.5 Day)

3.12 Evaluation of BR expression by Coomassie Brilliant blue-stained SDS-PAGE and Western blot analysis (0.5 Day)

3.13 Iterative clone-picking to optimize expression of His_10_-tagged bacteriorhodopsin (2–3 Weeks per iteration)

3.14 Large-volume cultivation of a selected transformed *H. salinarum* L33 clone (2 Weeks)

3.15 Purple membrane preparation (1.5 Days)

3.16 Detergent-mediated reconstitution of BR into preformed liposomes (2 Days)

3.17 Functional characterization of BR proteoliposomes by photoactivity assay (1 Day)

3.18 Structural characterization of purple membranes by negative stain transmission electron microscopy (1 Day)

### 2.1. Materials

- Phusion High-Fidelity PCR Kit (Thermo Fisher, Reinach, Switzerland; cat. no. F553L)
- 5′-pUC-BamHI-BR (forward primer): AAA AGG ATC CGA CGT GAA GAT GGG GC (Microsynth AG, Balgach, Switzerland; custom synthesis)
- 3′-BR-C-His_10_-HindIII (reverse primer): AAA AAA GCT TGA TTC AGT GGT GAT GAT GGT GAT GAT GGT GGT GAT GTC CGT CGC TGG TCG CGG CCG CGC (Microsynth AG, Balgach, Switzerland; custom synthesis)
- Primer for D96N mutation in BR-C-His10: CCG CTG TTG TTG TTA AAC CTC GCG TTG CTC GTT G (Microsynth AG, Balgach, Switzerland; custom synthesis)
- The plasmid pUC18-*bop*, i.e., the promotor and the gene of BR (*bop*) inserted into the commercially available pUC18 vector (New England BioLabs, Allschwil, Switzerland; cat. no. N3041)
- Gel extraction/PCR purification kit (NucleoSpin Gel and PCR Clean-up Kit, Macherey-Nagel, Oensingen, Switzerland; cat. no. 740609)
- DNA Ligation Kit (Thermo Fisher, Reinach, Switzerland; cat. no. K1423)
- E.Z.N.A. Plasmid Mini Kit I (VWR, Dietikon, Switzerland; cat. no. D6943-02)
- BamHI-HF (New England BioLabs, Allschwil, Switzerland; cat. no. R3136S)
- HindIII-HF (New England BioLabs, Allschwil, Switzerland; cat. no. R3104S)
- CutSmart buffer 10X (New England BioLabs, Allschwil, Switzerland; cat. no. B7204S)
- DH5α competent cells (Thermo Fisher, Reinach, Switzerland; cat. no. 18265017)
- plasmid pHS blue (shuttle vector: *E. coli* and *H. salinarum*) [39]. See Supplementary Information for nucleotide sequence (Material S1) and vector map (Figure S1)
- Agarose (Sigma-Aldrich, Buchs, Switzerland; cat. no. A9539)
- Tris (AppliChem, Bioswisstec, Schaffhausen, Switzerland; cat. no. A1430)
- Acetic acid (Sigma-Aldrich, Buchs, Switzerland; cat. no. 695092)
- Ethylenediaminetetraacetic acid (EDTA; Sigma-Aldrich, Buchs, Switzerland; cat. no. EDS)
- Luria-Bertani (LB) broth (VWR, Dietikon, Switzerland; cat. no. J106)
- Kanamycin sulfate (AppliChem, Bioswisstec, Schaffhausen, Switzerland; cat. no. 1493)
- Bacto^™^ Agar (BD, Allschwil, Switzerland; cat. no. 214010)
- E.Z.N.A. Plasmid Midi Kit (VWR, Dietikon, Switzerland; cat. no. D6904-03)
- Sodium chloride Ph.Eur/USP (NaCl; Schweizer Salinen, Pratteln, Switzerland; www.salz.ch, cat. no. 7731)
- Magnesium sulfate (MgSO_4_; Sigma-Aldrich, Buchs, Switzerland; cat. no. M2643)
- Trisodium citrate dihydrate (Merck, Zug, Switzerland; cat. no. 106448)
- Potassium chloride (KCl; Sigma-Aldrich, Buchs, Switzerland; cat. no. 60310)
- Bacteriological peptone (OXOID, Pratteln, Switzerland; cat. no. LP0037)
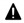
**CRITICAL** Other peptone products might not work optimally.
- Sodium hydroxide (NaOH; Sigma-Aldrich, Buchs, Switzerland; cat. no. S8045)
- Milli-Q ultrapure water (from Millipore water system)
- Deionized water from in-house system
- *H. salinarum* strain S9 [65]
- *H. salinarum* strain L33 [53,66]
- Novobiocin sodium salt (Sigma-Aldrich, Buchs, Switzerland; cat. no. N1628)
- Hydrochloric acid (HCl) fuming, ≥37% (Sigma-Aldrich, Buchs, Switzerland; cat. no. 30721) ‘CAUTION’ Highly corrosive. Work in a fume hood and wear goggles and protective clothing.
- Polyethylene glycol 600 (PEG600; Sigma-Aldrich, Buchs, Switzerland; cat. no. 87333)
- Sucrose (Sigma-Aldrich, Buchs, Switzerland; cat. no. S7903)
- Calcium chloride dihydrate (CaCl_2_; Merck, Zug, Switzerland; cat. no. 102382)
- Deoxyribonuclease I from bovine pancreas (DNase; Sigma-Aldrich, Buchs, Switzerland; cat. no. DN25)
- Sodium azide (NaN_3_; AppliChem, Bioswisstec, Schaffhausen, Switzerland; cat. no. A1430) ‘CAUTION’ Highly toxic substance. Needs special handling and waste disposal.
- Coomassie Brilliant blue R-250 (AppliChem, Bioswisstec, Schaffhausen, Switzerland; cat. no. A1092)
- Immobilon-P polyvinylidene difluoride (PVDF) transfer membrane (Merck Millipore Ltd., Zug, Switzerland; cat. no. IPVH00010)
- Albumin from bovine serum (BSA, Sigma-Aldrich, Buchs, Switzerland; cat. no. A3912)
- Tween 20 (AppliChem, Bioswisstec, Schaffhausen, Switzerland; cat. no. A4974)
- Anti-penta-His mouse primary antibody (Qiagen, Hombrechtikon, Switzerland; cat. no. 34660)
- Goat anti-mouse horseradish peroxidase (HRP)-conjugated secondary antibody (Bio-Rad, Cressier, Switzerland; cat. no. 172-1011)
- Amersham ECL Western Blotting Detection Reagents (GE Healthcare, Opfikon, Switzerland; cat. no. RPN2209)
- Super RX-N Fuji Medical X-ray film (Fujifilm, Dielsdorf, Switzerland; cat. no. 47,410 19284)
- Glycerol (Sigma-Aldrich, Buchs, Switzerland; cat. no. 49770)
- 1,2-dioleoyl-sn-glycero-3-phosphocholine (DOPC) dissolved in chloroform (Avanti Polar Lipids, Alabaster, AL, USA; cat. no. 850375)
- Nitrogen (or any other inert) gas
- n-octyl-β-D-glucopyranoside (OG, Glycon Biochemicals, Luckenwalde, Germany; cat. no. D97001)
- Potassium phosphate monobasic (KH_2_PO_4_, Sigma-Aldrich, Buchs, Switzerland; cat. no. P5655)
- Potassium phosphate dibasic (K_2_HPO_4_, Sigma-Aldrich, Buchs, Switzerland; cat. no. P3786)
- Uranyl formate (Thomas Scientific, cat. no. C993L42). ‘CAUTION’ Highly toxic substance. Needs special handling and waste disposal according to radioactivity regulations.

### 2.2. Equipment

- PCR cycler: we use a SensoQuest Labcycler
- Agarose gel electrophoresis apparatus
- pH meter: we use a Seven Compact pH meter (Mettler Toledo, cat. no. 30019028)
- Filters for media sterilization: we use Jet Biofil Filter Upper Cups 0.22 µm (Axonlab, cat. no. FPE-214-000)
- Incubator for liquid cultures: we use an Infors HT Multitron Standard incubator
- Spectrophotometer for liquid cultures: we use a Pharmacia Biotech Ultraspec 3000 UV-visible spectrophotometer
- Light microscope: we use a Leitz Laborlux 11 light microscope
- Incubator for agar plates: we use a Memmert TNB400 incubator
- Centrifuge for 15 mL and 50 mL conical vials: we use a Beckman Coulter Allegra X-15R centrifuge
- Centrifuge for 1.5 mL and 2 mL test tubes: we use an Eppendorf Centrifuge 5424R
- Ultracentrifuge for 1 mL tubes: we use a Beckman Coulter OptimaTM MAX-XP ultracentrifuge
- For membrane homogenization we use a glass homogenizer size 19 (1 mL) from Kontes Glass Co
- For chromophore-based protein quantification we use a NanoDrop 1000 spectrophotometer (Thermo Fisher, Reinach, Switzerland)
- SDS-PAGE apparatus: we use a Bio-Rad Mini-PROTEAN Tetra System
- Blotting apparatus for Western blot: we use a Bio-Rad TransBlot SD Semi-Dry Transfer Cell
- Centrifuge for 1 L bottles: we use a Herolab HiCen XL centrifuge
- Ultracentrifuge for 94 mL tubes: we use a Beckman Coulter OptimaTM L-90K ultracentrifuge
- 10 mL round bottom flasks with glass stoppers and suitable holder
- Desiccator equipped with a vacuum pump: we use a Nalgene plastic desiccator (Thermo Fisher, Reinach, Switzerland)
- Thermoshaker for 1–2 mL sample tubes: we use an Eppendorf ThermoMixer C
- Extruder with heating block and two gas tight syringes: we use an Avanti Mini-Extruder (Avanti cat. no. 610000) and two 1 mL gas tight syringes (Avanti, cat. no. 610017)
- Membranes for extruder: we use polycarbonate membranes with pore size 0.2 μm (Avanti, cat. no. 610006)
- Filter supports for extruder: we use 10 mm filter supports (Avanti, cat. no. 610014)
- Dialysis tubing: we use 10 mm Membra-Cel dialysis tubing with molecular weight cut-off 14 kDa (Membra-Cel, Roth, cat no. 1780.1)
- Plastic clips: we use Spectrum Dialysis Tubing Closures (Spectra/Por, cat. no. 888-11614)
- 2 L beaker or Erlenmeyer flask
- Unautoclaved 2 mL sample tubes
- Magnetic stirrer and magnetic stir bar
- Temperature regulated water bath with connective tubing and a glass cooling cell: we use a Julabo F10 ultrapure water bath (Gemini, cat. no. 01819) and a SONOPULS KG 3 (Faust, cat. no. 9.650 235) glass cooling cell with water jacket
- Micro pH electrode with integrated temperature sensor: we use an InLab Micro Pro-ISM electrode (Mettler Toledo, cat. no. 51344163)
- Computer with LabX direct pH 2.3 software (Mettler Toledo)
- Warm white LED lamp: we use a JANSÖ LED lamp (IKEA, cat. no. 903.860.12)
- Laboratory film: we use Parafilm M sealing film
- Syringe and filter: we use disposable 10 mL syringes ONCE/CODAN (HUBERLAB, cat. no. 3.7410.06) and single use hydrophilic 0.2 μm Sartorius Minisart syringe filters (fisher scientific, cat. no. 10730792)
- Carbon-coated copper electron microscopy grids: we use 400 mesh copper grids with a thin carbon film (Gridtech, cat. no. Cu-400CN)
- Microscope slide
- Glow discharge system including a high voltage power supply and a vacuum chamber: we use a custom build but any commercially available system works
- Precision forceps: we use 0.1 × 0.06 mm Dumont Dumoxel Tweezers #5 (World Precision Instruments, cat. no. 14098)
- Blotting paper for microscope slides: we use Macherey-Nagel MN 224 blotting paper for microscope slides
- Electron microscopy grid storage box: we use a grid storage box for 100 grids from Electron Microscopy Sciences
- Transmission electron microscope: we use a FEI Tecnai Spirit transmission electron microscope (Thermo Fisher, Reinach, Switzerland) equipped with an FEI Eagle CCD camera

## 3. Procedure

### 3.1. Site-Directed Mutagenesis of Bop in pUC18 (0.5 Day)

- Introduce a C-terminal His_10_-tag into the *bop* gene, which encodes wild-type BR (BR-wt), by PCR with pUC18-*bop* as a template, 5′-pUC-BamHI-BR as a forward primer and 3′-BR-C-His_10_-HindIII as a reverse primer (see Section 2.1). You can use a PCR kit of your choice, we use the Phusion High-Fidelity PCR kit. Prepare the following PCR master mix (Table 1) on ice or follow the manufacturer’s instructions for your preferred kit:
- Run the PCR reaction using the following program (Table 2):
- Purify your PCR product (*bop-C-His_10_*) using a PCR purification kit (see Section 2.1) and follow the manufacturer’s instructions. We use an elution volume of 32 µL.

### 3.2. Insertion of Bop-C-His_10_ into pHS Blue (0.5 Day)

- Digest the purified PCR product with BamHI-HF and HindIII-HF. Prepare the following reaction mixture (Table 3) and incubate for 1.5 h at 37 °C.
- In parallel, digest the pHS blue plasmid with BamHI-HF and HindIII-HF as well. Prepare the following reaction mixture (Table 4) and incubate for 1.5 h at 37 °C.
- Load the digested insert and the digested pHS blue plasmid on a 1% (*w*/*v*) agarose gel prepared with TAE buffer and let it run.
- Cut out the resulting bands for the insert and the plasmid and purify them using a gel extraction kit (see Section 2.1). Elute with a volume of 20 µL.
- Ligate the plasmid and the insert using a DNA ligation kit of your choice (see Section 2.1). Prepare the following ligation reaction mixture (Table 5) and incubate it for 45 min at RT.

### 3.3. Amplification of pHS Blue-Bop-C-His_10_ in E. coli DH5α (1 Week)

- Let 50 µL of *E. coli* DH5α competent cells thaw on ice.
- Add 5 µL of the ligation product (pHS blue plasmid with *bop-C-His_10_* insert) to the competent cells. Incubate on ice for 20 min.
- Incubate for 60 s at 42 °C.
- Incubate for 2 min on ice.
- Add 900 µL of LB and incubate at 700 rpm for 30 min at 37 °C in an Eppendorf tube shaker.
- Centrifuge at 14,000 rpm for 2 min at RT.
- Remove 880 µL of the supernatant.
- Resuspend the pellet in the remaining supernatant.
- Spread out the transformed cells on an LB-agar plate supplemented with 25 µg/mL kanamycin. Incubate overnight at 37 °C.
- Pick 4 colonies from the LB-agar plate and, with each, inoculate 10 mL of LB medium supplemented with 25 µg/mL kanamycin. Incubate at 180 rpm overnight at 37 °C.
- Use 5 mL of the overnight cultures to prepare glycerol stocks for each colony at a final concentration of 20% glycerol.
- Isolate the pHS blue-*bop-C-His_10_* plasmid from the remaining 5 mL of the overnight cultures of each colony using a plasmid DNA isolation kit of your choice. We use an E.Z.N.A. Plasmid DNA Mini Kit I with an elution volume of 60 µL.
- Measure the concentration of the isolated pHS blue plasmid by UV-Vis spectroscopy at 260 nm. The OD_260_/OD_280_ ratio should be between 1.8 and 2.0, indicating pure DNA.
- Digest the isolated plasmids with BamHI-HF and HindIII-HF. Prepare the following reaction mixture (Table 6) and incubate for 1.5 h at 37 °C.
- Load the digested plasmid on a 0.8% (*w*/*v*) agarose gel prepared with TAE buffer and run the gel.
- The gel should show two bands, one for the vector (~10.7 kb) and one for the insert (~1.2 kb). If that is the case, let the DNA of bacteriorhodopsin in the pHS blue-*bop-C-His_10_* construct be sequenced by a service company and check for your desired modification/mutation, and possible random mutations. If the sequence of a clone is correct, inoculate 50 mL LB supplemented with 25 µg/mL kanamycin with 5 µL glycerol stock of the corresponding clone and incubate at 180 rpm overnight at 37 °C.
- Isolate the pHS blue-*bop-C-His_10_* plasmid from the overnight culture using a plasmid DNA isolation kit of your choice. We use an E.Z.N.A. Plasmid DNA Midi Kit I with an elution volume of 500 µL. Measure the DNA concentration as described above.

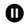
**PAUSE STEP** pHS blue-*bop-C-His_10_* plasmids can be stored at −20 °C for years.

### 3.4. Preparation of H. salinarum L33 Cells for Transformation (2–3 Days)

- Inoculate 10 mL L37 medium in a 50 mL conical tube with 10 µL *H. salinarum* L33 from a glycerol stock. This suffices for four transformations.
- Incubate the culture at 170 rpm and 40 °C until the OD_600_ is between 0.6 and 0.8, typically reached after 2–3 days.

### 3.5. Preparation of Plasmid for Transformation (10 Min)

- Place a volume containing 3 µg of the pHS blue-*bop-C-His_10_* plasmid at the bottom of a 15 mL conical vial, add 5 M NaCl to the drop to reach a final concentration of 2 M (e.g., 3 µL plasmid (1 µg/µL) + 2 µL 5 M NaCl) and mix the resulting drop with a pipette. Close the tube to avoid evaporation and store at room temperature until it is used for transformation.

### 3.6. Transformation of H. salinarum L33 (2 H)

**Figure 1:**
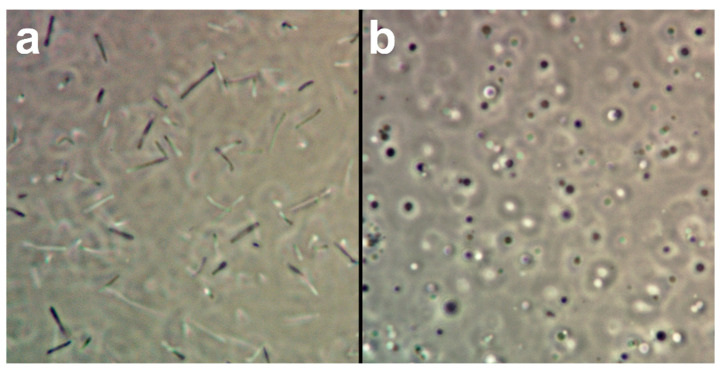
*H. salinarum* L33 cells as rods and spheroplasts. Micrographs of *H. salinarum* L33 cells before (**a**) and after supplementation of ethylenediaminetetraacetic acid (EDTA) to a final concentration of 25 mM (**b**). (**a**) Before the addition of EDTA, the cells are motile rods up to a length of 6 µm. Rarely, mitotic cells with spherical shape can be found. (**b**) After the addition of EDTA, the archaeal cell envelope, the S-layer, is decomposed and all cells adopt a spherical shape in which they are deprived of autonomous motion. Shown are excerpts of 50 × 50 µm.

- Pellet the *H. salinarum* culture at 4000× *g* for 15 min at room temperature, discard the supernatant, resuspend the pellet in 1 mL of SPH and take a 50 µL sample for spheroplast formation control.
- Add 50 µL of EDTA solution to the resuspended cells, gently mix by shaking the tube by hand and take another 50 µL sample.
- Check the formation of spheroplasts by comparing the 50 µL samples before and after EDTA addition in a light microscope. Normal *H. salinarum* L33 cells are motile rods (Figure 1a), while spheroplasts are round and only move by Brownian motion (Figure 1b).
- If all cells turned from rods to spheroplasts, add 200 µL of the resuspended cells to the 15 mL conical vial containing the plasmid and incubate for 20 min at room temperature.
- Carefully add an equal volume of PEG solution to the spheroplast-DNA-mix (200 µL spheroplasts + volume of diluted DNA, e.g., 5 µL) and incubate for 20 min at room temperature.
- Fill up the conical vial to 10 mL with spheroplast dilution solution and incubate for 30 min at 40 °C.

### 3.7. Outgrowth of Transformed Cells (2 Days)

- Pellet the cells at 4000× *g* for 10 min at room temperature and resuspend in 1 mL regeneration medium. Place the tube horizontally in the incubator instead of standing in a rack and incubate at 180 rpm and 37 °C for 2 days.

### 3.8. Plating and Colony Formation (1–2 Weeks)

- Dilute the transformed cells and spread out 50 µL on L37-agar plates supplemented with 0.3 µg/mL novobiocin. As transformation efficiency varies strongly, try different dilutions (e.g., 1:100 and 1:1000). Seal the plates in plastic bags or with plastic tape to avoid evaporation during incubation (see Section 5.2). Incubate at 37 °C for 1–2 weeks.

### 3.9. Colony Picking and Cultivation (1 Week)

**Figure 2:**
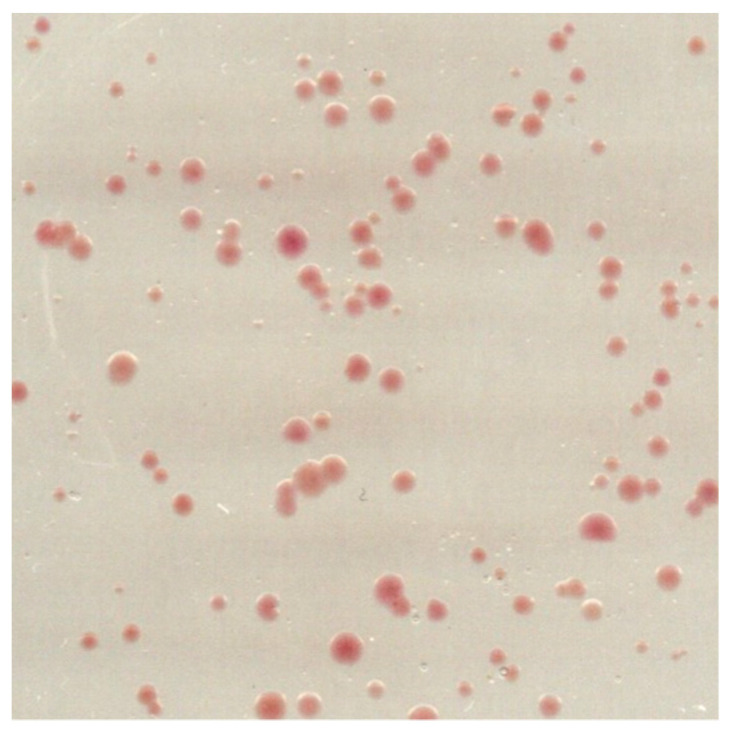
L37-agar plate of *H. salinarum* L33 transformed with BR-C-His_10_. A total of 50 µL of *H. salinarum* L33 cells transformed with BR-C-His_10_ was plated out after 2 days of outgrowth in a dilution of 1:100 and incubated at 37 °C for two weeks. Shown is an excerpt of the plate 4 × 4 cm in size.

- Pick 10–20 colonies from the L37-agar plates (Figure 2) and with each, inoculate 20 mL of L37 supplemented with 0.3 µg/mL novobiocin in a 50 mL conical vial. Incubate at 170 rpm and 40 °C for 1 week.
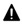
**CRITICAL STEP** Try to pick colonies of different sizes and colour intensities. Colonies with a strong red colour may express considerable amounts of BR-wt. In our specific case, the colours did not vary significantly and the size of the clones did not correlate with the ratio of BR-C-His_10_ to BR-wt.

### 3.10. Glycerol Stock Preparation (30 Min)

- Centrifuge 8 mL of each culture at 4000× *g* for 15 min at room temperature and resuspend in 3.2 mL L37. Add 800 µL of 50% (*w*/*v*) glycerol solution to reach a final concentration of 10%. Freeze with liquid nitrogen and store at −80 °C.

### 3.11. Clone Screening by Small-Volume Purple Membrane Preparation (0.5 Day)

- Centrifuge 10 mL of each culture at 5000× *g* for 15 min at 4 °C, resuspend in 1.6 mL basal salt and transfer the sample to a 2 mL Eppendorf tube. Centrifuge at 8000× *g* for 5 min at 4 °C and discard the supernatant.
- Add 700 µL of 10 mM MgSO_4_ supplemented with 30 µg DNAse (=12 µL of a 2.5 mg/mL solution). Lyse the cells by resuspending with a pipette until the solution is homogeneous.
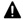
**CRITICAL STEP** Remaining DNA leads to a viscous, inhomogeneous purple membrane sample and reduces the yield.
- Remove the cell debris by centrifuging the samples at 4300× *g* for 1 min at 4 °C, followed by two centrifugations at 7000× *g* for 1 min at 4 °C. Transfer the supernatant to a new tube after each centrifugation.
- Transfer the samples to 1 mL ultracentrifuge tubes and ultracentrifuge the membranes at 55,000× *g* for 15 min at 4 °C. Resuspend the pellets in 700 µL Milli-Q ultrapure water with a pipette and repeat the last centrifugation.
- Resuspend the pellets in 700 µL Milli-Q ultrapure water and ultracentrifuge the membranes at 60,000× *g* for 15 min at 4 °C. Resuspend the pellets in 700 µL Milli-Q ultrapure water with a pipette and repeat the last centrifugation.
- Resuspend the pellets in 25 µL Milli-Q ultrapure water with a pipette.
- Measure the concentration of bacteriorhodopsin in each sample by UV-Vis spectrometry (see Section 5.2).

### 3.12. Evaluation of BR Expression by Coomassie Brilliant Blue-Stained SDS-PAGE and Western blot analysis (0.5 Day)

**Figure 3:**
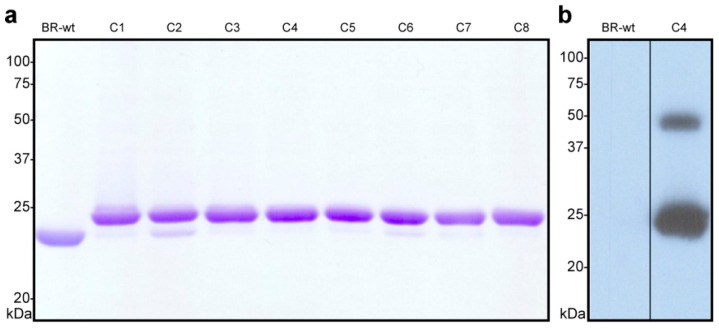
Analysis of BR-C-His_10_ clones by SDS-PAGE and Western blot. (**a**) Coomassie Brilliant blue-stained 13.5% SDS/polyacrylamide gel of isolated purple membranes from *H. salinarum* L33 clones transformed with BR-C-His_10_ (C1–C8) and BR-wt (isolated from *H. salinarum* S9) as a control. BR-C-His_10_ migrates slower compared to BR-wt because of the additional His_10_-tag. In each lane purple membranes corresponding to 5 µg of bacteriorhodopsin were loaded. (**b**) Western blot analysis of BR-wt and BR-C-His_10_ (C4) performed using an SDS gel run under similar conditions as in (**a**). The primary and secondary antibodies used were mouse monoclonal anti-penta-His antibody (dilution 1:3000) and horseradish peroxidase-conjugated goat anti-mouse antibody (dilution 1:3000), respectively. BR-wt showed no band, due to the absence of a His_10_-tag. BR-C-His_10_ reacted with the anti-penta-His antibody, confirming the presence of the introduced His_10_-tag. A weaker band around 50 kDa indicates the presence of SDS-resistant BR-C-His_10_ dimers.

- Load membranes of each sample corresponding to 5 µg of bacteriorhodopsin on a 13.5% SDS-PAGE gel. As a negative control, include an equivalent of *H. salinarum* S9 membranes corresponding to 5 µg of bacteriorhodopsin (for expression and purification of S9 membranes see Section 3.14 and Section 3.15 of this protocol). If available, as a positive control, include an equivalent of membranes from a *H. salinarum* strain, expressing a His_10_-tagged bacteriorhodopsin corresponding to 5 µg of bacteriorhodopsin. Run the electrophoresis at 200 V until the dye front reaches the end of the gel and stain the gel using Coomassie Brilliant blue R-250 according to standard protocols.
- For Western blot, load an equivalent of membranes of each sample corresponding to 0.5 µg of bacteriorhodopsin on a 13.5% SDS-PAGE gel. As a negative control, include an equivalent of *H. salinarum* S9 membranes corresponding to 0.5 µg of bacteriorhodopsin. Run the electrophoresis at 200 V.
- Transfer the samples onto a PVDF-membrane, e.g., using a semi-dry blotting system. Incubate the membrane in 30 mL TBS containing 3% (*w*/*v*) BSA for 1 h at room temperature under gentle agitation to reduce unspecific binding of the antibodies.
- Incubate the membrane with anti-penta-His mouse primary antibody diluted 1:3000 in 30 mL TBS containing 3% (*w*/*v*) BSA for 1 h at room temperature under gentle agitation.
- Wash the membrane three times with 30 mL TBS containing 0.5% (*v*/*v*) Tween 20 for 10 min at room temperature under gentle agitation. Incubate with goat anti-mouse horseradish peroxidase (HRP)-conjugated secondary antibody diluted 1:3000 in 30 mL TBS containing 3% (*w*/*v*) BSA for 1 h at room temperature under gentle agitation.
- Wash the membrane three times with 30 mL TBS containing 0.5% (*v*/*v*) Tween 20 for 10 min at room temperature under gentle agitation.
- Add 2 mL of ECL-detection reagent (1 mL of each) to the membrane and incubate for 2 min at room temperature under gentle agitation. Seal the membrane in a plastic bag and expose an X-ray film.
- Estimate the ratio of His_10_-tagged to wild-type bacteriorhodopsin in the different samples by SDS-PAGE (Figure 3a) and identify the best clone(s). If a pure clone could be obtained (i.e., no or a very small lower band), continue working with the glycerol stock of the corresponding clone. As an example, in Figure 3a, clone number 2 (C2) contains a considerable amount of BR-wt, while clone number 4 (C4) can be considered of high purity. The Western blot in Figure 3b shows that clone C4 contains the engineered C-terminal His_10_-tag. A smaller band around 50 kDa indicates the presence of SDS-resistant BR-C-His_10_ dimers.

### 3.13. Iterative Clone-Picking to Optimize Expression of Engineered BR (2–3 Weeks)

- If the ratio of His_10_-tagged to wild-type bacteriorhodopsin of the best clone is not satisfactory, inoculate 20 mL of L37 supplemented with 0.3 µg/mL novobiocin in a 50 mL conical vial with 10 µL of the glycerol stock of the best clone and incubate at 170 rpm for 1 week at 40 °C. Prepare from this culture new L37-agar plates, pick clones and repeat the analysis as described in Section 3.8, Section 3.9, Section 3.10, Section 3.11 and Section 3.12. This process may have to be iterated several times to obtain a clone that only expresses the His_10_-tagged bacteriorhodopsin construct.

### 3.14. Large-Volume Cultivation of a Selected Transformed H. Salinarum L33 Clone (2 Weeks)

- Inoculate 50 mL of L37 supplemented with 0.3 µg/mL novobiocin in a 250 mL Erlenmeyer flask with 50 µL of the glycerol stock of the selected clone. Incubate at 170 rpm for 1 week at 40 °C.
- Inoculate 2 L of L37 supplemented with 0.3 µg/mL novobiocin in a 5 L Erlenmeyer flask with 40 mL of the previous culture. Incubate at 170 rpm for 1 week at 40 °C. For a standard large-volume cultivation, we inoculate three flasks containing 2 L of each medium.

### 3.15. Purple Membrane Preparation (1.5 Days)

- Centrifuge the culture at 10,000× *g* for 20 min at 4 °C, resuspend the pellet in 400 mL basal salt using a 25 mL serological pipette and repeat the last centrifugation. Before centrifugation, measure the weight of the empty centrifugation tubes to determine the cell mass.
- Resuspend the pellet in 25 mL/g cell mass of Milli-Q ultrapurified water supplemented with 0.02% (*w*/*v*) NaN_3_ (preservative to inhibit bacterial growth). Add 1 mg DNase/g cell mass and gently stir the solution at 4 °C overnight to lyse the cells by osmotic shock.
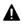
**CRITICAL STEP** Remaining DNA leads to a viscous, inhomogeneous purple membrane sample and reduces the yield.
- Remove the cell debris by centrifuging the sample at 4300× *g* for 10 min at 4 °C, followed by two centrifugations at 7600× *g* for 10 min at 4 °C. Transfer the supernatant to a new tube after each centrifugation. Over the course of the three centrifugations, the cell debris pellets should change in color from brown to purple.
- Transfer the supernatant to two 94 mL ultracentrifuge tubes, fill them up with Milli-Q ultrapurified water and ultracentrifuge at 55,000× *g* for 1 h at 4 °C. Resuspend the pellets in Milli-Q ultrapurified water with a pipette, combine them in one 94 mL ultracentrifuge tube, fill it up with Milli-Q ultrapurified water and repeat the last centrifugation.
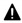
**CRITICAL STEP** There might still be some remaining cell debris as a gray spot at the center of the pellets. Resuspend the purple parts around it very carefully with a pipette by letting the water flow over the pellet without detaching the gray part from the wall of the tube. If the gray spots are relatively small, the whole pellets can be resuspended after the first centrifugation. It will be easier to separate the cell debris after the second centrifugation when the two gray spots from the previous pellets are combined in one spot.
- Resuspend the pellet in Milli-Q ultrapurified water in the same tube with a pipette and ultracentrifuge at 60,000× *g* for 1 h at 4 °C. Resuspend the pellet with Milli-Q ultrapurified water in the same tube and repeat the last centrifugation.
- Resuspend the pellet in ~0.5 mL/g cell mass of Milli-Q ultrapurified water supplemented with 0.02% (*w*/*v*) NaN_3_ with a pipette and homogenize using a glass homogenizer. You may have to adjust the volume depending on the expression efficiency of your construct and the concentration needed for following experiments.
- Measure the concentration of bacteriorhodopsin in your sample by UV-Vis spectrometry (see Section 5.2).
- Determine the purity of your sample and validate the presence of the His_10_-tag by Coomassie Brilliant blue-stained SDS-PAGE and Western blot (see Section 3.12).
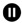
**PAUSE STEP** Purple membrane samples in 0.02% NaN_3_ can be stored at 4 °C for years.

### 3.16. Detergent-Mediated Reconstitution of BR into Preformed Liposomes (2 Days)

- Dry 10 mg of DOPC dissolved in chloroform in a 10 mL round bottom flask under a constant stream of nitrogen gas with gentle shaking. A thin layer of lipid is beneficial for the optimal formation of liposomes. Remove residual chloroform traces by keeping the flask under vacuum in a desiccator overnight.
- On the next day, solubilize 400 μg of BR in 3% (*w*/*v*) OG at a protein concentration of 1 mg/mL (e.g., add 120 μL of 10% (*w*/*v*) OG to 100 μL 4 mg/mL BR and 180 μL ultrapure water). Incubate the mixture for 3 h at room temperature under gentle rotation or shaking (avoid foam formation) and ensure that it is protected from light. Finally, the mixture is centrifuged for 15 min at 100,000× *g* to remove unsolubilized material.
- In the meantime, add 2 mL of hydration buffer to the dried lipid and shake it for around 30 min until the lipid is completely resuspended.
- Assemble the extruder with a 200 nm pore size membrane and one filter support on each side. Flush it with 1 mL of hydration buffer using the syringes to pass the solution back and forth. Then, pass the liposome suspension through in the same way 19 times. With an increasing number of passages, the suspension should become more transparent, indicating a reduction in particle size.
- Start this step before the centrifugation of solubilized BR. Destabilize the extruded liposomes with 0.4% (*w*/*v*) OG by adding 83 μL of 10% (*w*/*v*) OG for 15 min with gentle shaking.
- Add the solubilized BR to the destabilized liposomes and incubate with gentle shaking for 30 min.
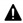
**CRITICAL STEP** The final OG concentration after adding the solubilized protein to the liposomes should always be close to 0.8%. This ensures optimal destabilization and the most efficient protein insertion. Should you want to change the amount of protein for the reconstitution, make sure to compensate the amount of detergent added.
- In the meantime, soak the dialysis tubing and rinse it with Milli-Q ultrapure water.
- Close the dialysis tubing on one side with a plastic clip, fill in the protein liposome mixture and then close the other side with a second plastic clip. Place the filled dialysis tube in 2 L of dialysis buffer. Add a magnetic stir bar and dialyse overnight at 4 °C with gentle stirring. Up to two samples can be dialysed together in 2 L of dialysis buffer.
- Harvest the dialysed samples and ultracentrifuge them 10 min at 200,000× *g*. Resuspend the pellet in 1 mL of measuring solution by pipetting and centrifuge again. Wash the samples once more by repeating the resuspension and centrifugation, and finally resuspend the proteoliposomes in 800 μL of measuring solution.

### 3.17. Functional Characterization of BR Proteoliposomes by Photoactivity Assay (1 Day)

**Figure 4:**
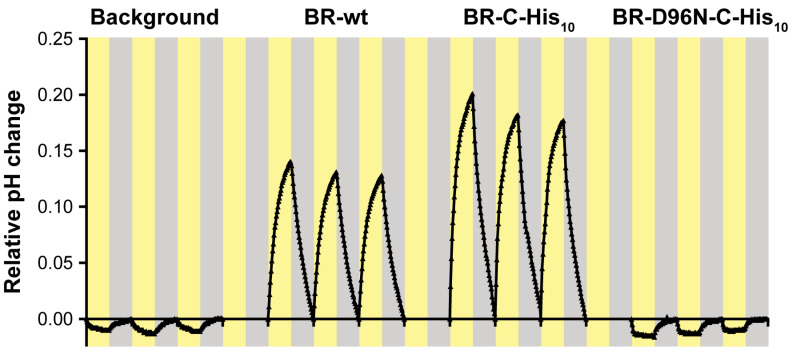
Photoactivity of BR versions reconstituted into liposomes. Drift-corrected photoactivity measurements of measuring solution (Background) or BR-wt, BR-C-His_10_ and BR-D98N-C-His_10_ proteoliposomes. Yellow and gray shading indicate 15 min light and dark cycles. The pH of the extracellular solution of proteoliposomes containing functional BR versions (wt and C-His_10_) increases upon illumination, demonstrating the proton pumping activity and thus functional insertion. In contrast BR-D96N-C-His_10_ proteoliposomes exhibit only background level pH change upon illumination since D96N is a BR version with defective proton pumping activity. All proteoliposomes were reconstituted with the same amount of protein; thus, the increased pH change of the His-tagged version is either due to higher protein activity or more efficient reconstitution.

- Transfer the proteoliposome suspension to a clear unautoclaved 2 mL tube and add an appropriately sized magnetic stir bar. Autoclaved tubes tend to get cloudy, thus reducing the illumination of the samples in the next step.
- The photoactivity of the proteoliposomes is assessed by measuring the pH change in the extravesicular medium upon illumination using a micro pH electrode. The sample should be constantly stirred and cooled to 18 °C by placing the tube in a transparent glass cooling cell with a water jacket. Illumination is provided by a warm white LED lamp (i.e., full spectrum). Data are collected in intervals of 30 s using a computer connected to the pH meter and running an appropriate pH meter software. Optimal illumination cycles for the described proteoliposomes comprise an initial 15 min adaptation period in the dark, followed by alternating periods of illumination and darkness of 15 min each (see Figure 4). Make sure to collect at least 4 peaks as the first one might exhibit unfavourable fluctuations. For a detailed overview and an illustration of the setup, see Hirschi et. al. (2019) [67].
- Finally, the data can be corrected for continuous pH drift by making the following approximation. A continuous piecewise linear function is constructed from the starting points of each illumination cycle and subtracted from the pH curve. This will set the starting points of each cycle to 0, thus converting the scale to relative pH change and removing any drift occurring during the recording. For a more detailed description of this process consult Harder et. al. (2016) [68].

### 3.18. Structural Characterization of Purple Membranes by Negative Stain Transmission Electron Microscopy (1 Day)

**Figure 5:**
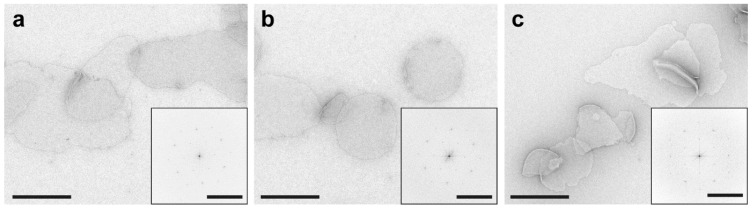
Structural characterization of purple membranes by negative stain transmission electron microscopy. Electron micrographs of negatively stained purple membrane patches containing BR-wt (**a**), BR-C-His_10_ (**b**) and BR-D96N-C-His_10_ (**c**). Insets show the power spectra of selected areas at higher magnification and demonstrate the crystallinity of the membrane patches. Scale bars represent 500 Å and 50 Å^−1^ in the insets.

- Prepare 0.75% (w/v) uranyl formate solution and filter it with a 0.2 μm syringe filter before use to remove any precipitates.
- Carbon-coated copper grids are placed with the coated (shiny) side facing up on a Parafilm-wrapped microscope slide. Use precision forceps while handling microscope grids and only hold them on the edges to avoid damaging the center. Grids are then glow discharged for 10 s under vacuum.
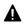
**CRITICAL STEP** Glow discharging electron microscopy grids is essential for the efficient adsorption of hydrophilic samples such as proteoliposomes to the hydrophobic grids. However, excessive glow discharging should be avoided as it can damage the extremely thin carbon layer.
- A total of 5 μL of purple membrane at a concentration of about 1 mg/mL is adsorbed onto a glow discharged grid for 1 min.
- The liquid is removed by gently touching the side of the grid with blotting paper. Then, the grid is immediately washed three times by placing a 5 μL drop of ultrapure water on the grid and removing it using the blotting paper.
- A total of 5 μL of uranyl formate solution is placed on the grid and immediately blotted. Then, another drop is added and incubated for 10 s before drying the grid again with blotting paper. Finally, let the grids air dry for a few minutes before placing them in a grid storage box.
- The general procedure of operating a transmission electron microscope can be obtained from the operating manual. The size and morphology of the purple membrane patches (see Figure 5) can be assessed at low magnification (e.g., 5–10,000×). The structural integrity and organization of the BR crystals can be assessed by imaging the patches at higher magnification (e.g., 68,000×) and observing the power spectrum via the Fourier transformation of corresponding images (see Figure 5 insets).

## 4. Expected Results

We have presented a detailed protocol on the production of an engineered BR version having a C-terminal His_10_-tag (BR-C-His_10_). Furthermore, we have described the isolation procedure, and functional and structural analysis of the resulting PMs containing His-tagged BR. Importantly, this protocol opens the possibility to scientists to tailor own versions of BR for specific applications in basic research and applied sciences.

We first introduced a His_10_-tag at the C-terminus of the *bop* gene in the pUC18 vector and subsequently cloned the *bop-C-His_10_* gene into the pHS blue vector. *H. salinarum* L33 cells were then transformed with the resulting construct, and the PMs of several clones were isolated and analysed by SDS-PAGE (Figure 3). The BR-deficient *H. salinarum* L33 [53,54] strain has the tendency to produce revertants via homologous recombination between the transformed *bop* gene copy and the native *bop* gene [46,69], which is here partially disrupted by an insertion element [54]. This might result in purple membranes containing mixtures of BR-wt and the desired engineered version, e.g., BR-C-His_10_ (Figure 3). To avoid this, rounds of clone optimization as described in the protocol are necessary and were applied to obtain a clone producing maximal amounts of His-tagged BR (Figure 3). After the successful identification of a suitable clone, larger cultures were grown and the isolated PMs were biochemically, functionally and structurally analysed.

BR-C-His_10_ and BR-wt as control were reconstituted using DOPC lipids and their functions studied using a photoactivity assay. Upon illumination, BRs incorporated in the proteoliposomes pump protons into the lumen, which can be detected in the surrounding medium using a micro pH-electrode (Figure 4). Proteoliposomes containing BR-C-His_10_ or BR-wt produced a pH-gradient of comparable amplitude upon illumination, which decays when the illumination is stopped. This shows that the C-terminal His_10_-tag is not interfering with the proton pumping function of BR. Transmission electron microscopy analysis of PMs containing BR-C-His_10_ (Figure 5b) shows comparable sizes and shapes of membrane patches as for PMs containing BR-wt (Figure 5a). Power spectra calculated from electron micrographs indicated that His-tagged PM patches possess the same highly ordered hexagonal 2D crystal structure (Figure 5b, inset) as PMs containing BR-wt (Figure 5a, inset). This demonstrates that the introduction of a C-terminal His_10_-tag is not interfering with the formation of the typical PM structure.

**Figure 6:**
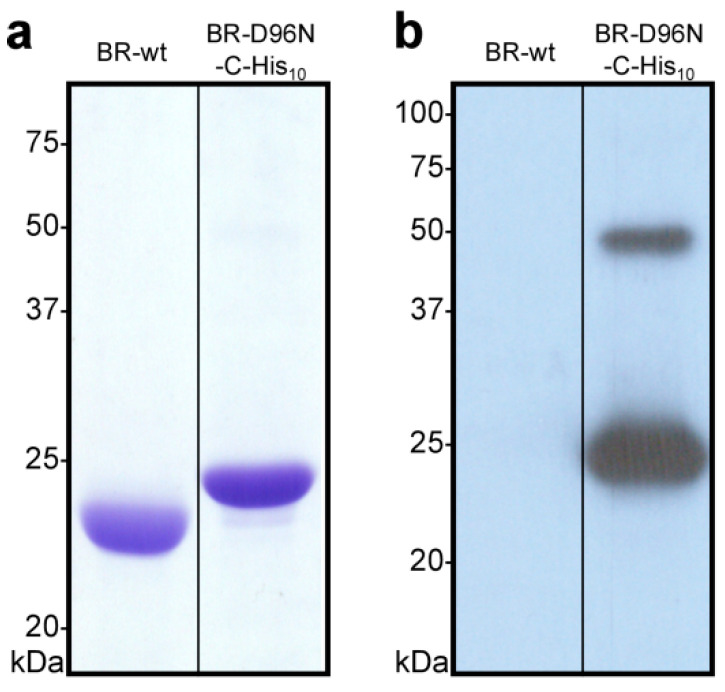
Analysis of BR-D96N-C-His_10_ by SDS-PAGE and Western blotting. (**a**) Coomassie Brilliant blue-stained 13.5% SDS/polyacrylamide gel of isolated purple membranes from *H. salinarum* L33 transformed with BR-D96N-C-His_10_ and BR-wt (isolated from *H. salinarum* S9) as a control. BR-D96N-C-His_10_ migrates slower compared to BR-wt, because of the additional His_10_-tag. In each lane, purple membranes corresponding to 10 µg of bacteriorhodopsin were loaded. (**b**) The Western blot analysis of BR-wt and BR-D96N-C-His_10_ performed under similar conditions as in (**a**). The primary and secondary antibodies used were mouse monoclonal anti-penta-His antibody (dilution 1:3000) and horseradish peroxidase-conjugated goat anti-mouse antibody (dilution 1:3000), respectively. BR-wt showed no band, due to the absence of a His_10_-tag. BR-D96N-C-His_10_ reacted with the anti-penta-His antibody, confirming the presence of the introduced His_10_-tag. A weaker band around 50 kDa, indicates the presence of SDS-resistant BR-D96N-C-His_10_ dimers [38,39].

Building on BR-C-His_10_, a functional mutant of BR that has a significantly prolonged M-state, i.e., ~1000-fold longer than that of BR-wt, was created by introducing a point mutation into the His-tagged construct (BR-D96N-C-His_10_) [37,70]. For such mutants, it is important to introduce the mutation into a BR version, which has a different molecular mass than the wild-type form, e.g., BR-C-His_10_, in order to be able to differentiate between mutant version and revertant wild-type BR by SDS-PAGE. The point mutation leading to a version of BR in which the aspartate at position 96 is exchanged for an asparagine, was introduced into the *bop-C-His_10_* gene in the pUC18 vector followed by cloning into the pHS blue vector. Next, *H. salinarum* L33 was transformed with the resulting construct (pHS blue BR-D96N-C-His_10_) and the PMs of several clones were isolated. Already after the first round of testing, a clone only expressing BR-D96N-C-His_10_, and no BR-wt, could be obtained. PMs of a larger culture of the selected clone were isolated, and the purity and integrity assessed by SDS-PAGE (Figure 6a) and Western blot analysis (Figure 6b). The former showed only one band at the expected size and no BR-wt band, while the later technique confirmed the presence of the introduced His-tag. A less prominent band around 50 kDa, indicated the presence of a minor fraction of SDS-resistant BR-D96N-C-His_10_ dimers [38,39]. This purple membrane preparation yielded 2.5 mg of BR-D96N-C-His_10_ (for BR quantifications refer to Section 5.2) per liter of cell culture. While yields can differ significantly between different constructs, yields of ~5 mg per liter of cell culture for wild-type BR from *H. salinarum* S9 and ~1–3 mg per liter of cell culture for mutants expressed in *H. salinarum* L33 can be expected.

Analogous to BR-wt and BR-C-His_10_, BR-D96N-C-His_10_ was reconstituted in DOPC lipids and its functional properties were analysed using the photoactivity assay described previously (Figure 4). In contrast to BR-C-His_10_, BR-D96N-C-His_10_ did not produce a pH change upon illumination. This confirms the expected slowing of the proton pumping activity by the introduction of the D96N mutation. To test, if the isolated mutant PMs form the same distinct patches and have the same 2D crystal structure, they were structurally analysed by negative stain transmission electron microscopy (Figure 5c). The electron micrographs of the PMs containing BR-D96N-C-His_10_ showed similar shapes and sizes of patches as the ones for PMs containing BR-wt (Figure 5a) and BR-C-His_10_ (Figure 5b). Analysis of calculated power spectra confirmed that the PMs retain their typical hexagonal 2D crystal lattice (Figure 5c, inset).

The obtained results from BR versions show that the presented protocol is applicable for the production of BR variants containing a purification tag or functional mutation. Importantly, the engineered modifications had no effect on the 2D assembly into PMs preserving this unique and highly valuable feature.

## 5. Setup

### 5.1. Reagent Setup

TAE buffer: Mix 40 mM Tris base, 20 mM acetic acid and 1 mM EDTA.

LB medium: Dissolve 25 g/L LB Broth powder in deionized water and autoclave.

LB-agar plates: Dissolve 25 g/L LB Broth powder in deionized water. Add 15 g/L Bacto™ Agar and autoclave.

L37 medium: Mix 10.2 mM trisodium citrate dihydrate, 4.3 M NaCl, 80 mM MgSO_4_, 27 mM KCl, and 10 g/L bacteriological peptone in deionized water. Adjust with NaOH to pH 7 at room temperature and sterilize for 2 h at 90 °C in a dry sterilizer.

Novobiocin stock solution: Dissolve 1 mg/mL novobiocin sodium salt in Milli-Q ultrapure water. Sterilize by filtration.

L37-agar plates: Mix 10.2 mM trisodium citrate dihydrate, 4.3 M NaCl, 80 mM MgSO_4_, 27 mM KCl, and 10 g/L bacteriological peptone in deionized water. Adjust with NaOH to pH 7 at room temperature. Add 15 g/L Bacto™ Agar and sterilize for 2 h at 90 °C in a dry sterilizer.

Spheroplast buffer (SPH): Mix 50 mM Tris-HCl (from 1 M stock solution at pH 8.75), 2 M NaCl, 27 mM KCl, and 150 g/L sucrose in Milli-Q ultrapure water. Sterilize by filtration.

EDTA solution: Dissolve 500 mM EDTA in SPH.

PEG solution: Mix 3 parts of PEG600 with 2 parts of SPH.

Spheroplast dilution solution: Mix 10.2 mM trisodium citrate dihydrate, 50 mM Tris-HCl (from 2 M stock solution adjusted to pH 7.4), 4.3 M NaCl, 80 mM MgSO_4_, 27 mM KCl, 1.55 mM CaCl_2_, and 150 g/L sucrose in Milli-Q ultrapure water. Sterilize by filtration.

Regeneration medium: Dissolve 150 g/L sucrose in L37 medium. Sterilize by filtration.

50% (*v*/*v*) glycerol solution: Mix glycerol and Milli-Q ultrapure water 1:1 and autoclave.

Basal salt: Mix 4.3 M NaCl, 80 mM MgSO_4_, and 27 mM KCl in deionized water and autoclave.

DNAse solution: Prepare a 2.5 mg/mL solution from the lyophilized DNAse using Milli-Q ultrapure water.

Tris-buffered saline (TBS): Mix 50 mM Tris and 150 mM NaCl in Milli-Q ultrapure water and adjust the pH to pH 8.

Hydration buffer: Mix 0.76 g/L KH_2_PO_4_, 2.51 g/L K_2_HPO_4_ and 100 mM KCl in Milli-Q ultrapure water and confirm the pH. If it is not 7.2, adjust accordingly.

Dialysis buffer: Mix 20 mM Tris and 150 mM NaCl in cold Milli-Q ultrapure water. Adjust the pH with HCl to 8 at 4 °C and fill up to 2 L with Milli-Q ultrapure water.

Measuring solution: Adjust a solution of 150 mM NaCl to pH 7.4 with NaOH and always check the pH immediately before use. Optionally, degas the solution with nitrogen gas to increase the pH stability.

### 5.2. Equipment Setup

L37-Agar plates: Due to the high sodium chloride concentration of L37-agar plates and the long incubation time (up to several weeks), it is necessary to seal the plates airtight, to prevent evaporation and salt crystal formation. This can be achieved by packing the plates into sealed plastic bags or by sealing the plates with several rounds of a standard plastic adhesive tape prior to incubation.

Chromophore-based protein quantification: To quantify the concentration of bacteriorhodopsin in a purple membrane suspension, the absorption at 568 nm is measured using a spectrophotometer. We use the UV-Vis program of a Thermo Fisher NanoDrop 1000 with measurements at 280 nm and 568 nm. High-quality purple membranes show a A_280_/A_568_ ratio of 2 [1] and lower. A higher ratio indicates the presence of proteins other than bacteriorhodopsin.

The concentration of bacteriorhodopsin in suspensions of purple membranes can be estimated by using a molar extinction coefficient of 63,000 L⋅mol^−1^⋅cm^−1^ at 568 nm [6].

## Figures and Tables

**Table 1:** PCR reaction mixture for site-directed mutagenesis of the *bop* gene.

Component	Amount (µL)	Final Concentration
pUC18-*bop* plasmid template	1	2 ng/µL
(100 ng/µL)		
5′-pUC-BamHI-BR forward primer	1	2 pM
(100 pM)		
3′-BR-C-His_10_-HindIII reverse primer	1	2 pM
(100 pM)		
dNTP’s (10 mM for each dNTP)	1	200 µM
Phusion CG buffer (5X)	10	1X
DMSO (100%)	1.5	3%
Phusion DNA Polymerase (2 U/µL)	0.5	0.02 U/µL
Nuclease-free water	34	
Total	50	

**Table 2:** PCR program for site-directed mutagenesis of the *bop* gene.

Cycles	Denaturation (94 °C)	Annealing (63 °C)	Elongation (72 °C)
1	2 min		
32	45 s	45 s	1 min
1			10 min

**Table 3:** Reaction mixture for the digestion of the PCR product.

Component	Amount (µL)	Final Concentration
Purified PCR product (350 ng/µL)	20	140 ng/µL
CutSmart buffer (10X)	5	1X
HindIII-HF (20 U/µL)	1	0.4 U/µL
BamHI-HF (20 U/µL)	1	0.4 U/µL
Nuclease-free water	23	
Total	50	

**Table 4:** Reaction mixture for the digestion of the pHS blue plasmid.

Component	Amount (µL)	Final Concentration
pHS blue plasmid (2 µg/µL)	7	280 ng/µL
CutSmart buffer (10X)	5	1X
HindIII-HF (20 U/µL)	3	1.2 U/µL
BamHI-HF (20 U/µL)	3	1.2 U/µL
Nuclease-free water	32	
Total	50	

**Table 5:** Reaction mixture for the ligation of pHS blue and the insert.

Component	Amount (µL)	Final Concentration
Digested pHS blue plasmid (130 ng/µL)	1	6.5 ng/µL
Digested insert (32 ng/µL)	14	22.5 ng/µL
Rapid ligation buffer (5X)	4	1X
T4 DNA ligase (5 U/µL)	1	0.25 U/µL
Total	20	

**Table 6:** Reaction mixture for the digestion of the amplified plasmid.

Component	Amount (µL)	Final Concentration
Isolated pHS blue-*bop-C-His_10_* (125 ng/µL)	8	100 ng/µL
CutSmart buffer (10X)	1	1X
HindIII-HF (20 U/µL)	0.5	1 U/µL
BamHI-HF (20 U/µL)	0.5	1 U/µL
Total	10	

## Supplementary Materials

The following are available online at https://www.mdpi.com/2409-9279/3/3/51/s1, Material S1: Nucleotide sequence of pHS blue, Figure S1: Vector map of pHS blue.
