# Supplementary material for: Engineering and Production of the Light-Driven Proton Pump Bacteriorhodopsin in 2D Crystals for Basic Research and Applied Technologies
Source: Methods Protoc. 2020 Jul 22;3(3):51. doi: 10.3390/mps3030051 (PMC7563565; doi:10.3390/mps3030051)
Supplement: Supplementary file 1 [file mps-03-00051-s001.pdf]

methods and protocols

MDPI

Supplementary Information for:

Engineering and Production of the Light-Driven Proton Pump Bacteriorhodopsin in 2D Crystals for Basic Research and Applied Technologies

Mirko Stauffer [1], Stephan Hirschi [1], Zöhre Ucurum [1], Daniel Harder [1], Ramona Schlesinger [2,*] and Dimitrios Fotiadis [1,*]

1. Institute of Biochemistry and Molecular Medicine, and Swiss National Centre of Competence in Research (NCCR) TransCure, University of Bern, 3012 Bern, Switzerland; mirko.stauffer@ibmm.unibe.ch(M.S.); stephan.hirschi@ibmm.unibe.ch (S.H.); zoehre.ucurum@ibmm.unibe.ch (Z.U.); daniel.harder@ibmm.unibe.ch (D.H.)
2. Department of Physics, Genetic Biophysics, Freie Universität Berlin, 14195 Berlin, Germany

* Correspondence: r.schlesinger@fu-berlin.de (R.S.); dimitrios.fotiadis@ibmm.unibe.ch (D.F.)

Material S1: Nucleotide sequence of pHS blue

```
CAATTCGCGGTCAAGAAAGACGGCCTTGACGTCGAGGTCAAGGGCGTCGAGGATACCGAGAAACTC
TGCGAGGACACTGCTGGCGGTGTCGCCGTCTTCGAGACGGCGCACCGCCAGCGTGTAGCGTTTGTT
CTTCACACGCGCGTAGAGTGTGGCGTAGGCGTGGAACGCAGTTGTTCCACGCTTCGCTTGTGAGTG
ATACAGGCCGTCTGTCCCGTCTTCATCGCCGTAGTAGGGCCGCAGGTGGAGGTCCGAAACGACCTC
CACCTGCTGGGGAAGGACGTCGAGAACGTCCTTCTGAAGGAGCGCGTTTCCGACTTGCTCAAGCGT
CTCAAGGTCGAATTTGGTGCGGAGATGGTAGAGAACCGAATTTTCGTGGGGGGCATCTTCGCTTTT
CTCACAGAGCGTTGAGACCGAGGTCCCGTCGGCGCACGCGCCGACGAGGACCTCGTAGATGTCTTC
AGCATCGATCTCGGCGTTTTCAGCGAGTGTGAGGGCGACTTCCTCGTCAAGAGCGTTGACGAGGAA
GTTAAGAAGCTGGTCCTCGTGGATTTCACCGTCTGCTTGCTGGGTTGTAGACACACCTTCAGCAAG
CAGACGTCTCAACTAACCGGCTTTGTGATGTACTGAAAATAAGCCTCGACGTGATCTTTCAGGCCA
AAATATTCTTCTTCTCCTTCATTGGCGTGGTTGTGCCAAGAGTTCGGAATTAGAATTATTCGACCG
TCTCCGGCCTCGGTTTTCGGATTGTATTGTCCACCATCTTCACAGTGTCCGCAAGTTCCGTCTCCG
TTGCCCTTACGACATTTATCTGAGCCCGGAATCTGAATGTATAGATTTCCATCGCTGTCGTAGAAG
AACCAATCCTTATACACATGACCAATCGTGTTGTTTCTCATACCAGTTCCGATCCCTAAGTGGACA
GACATGGCTTTGTCATCATTTGGATGCTCGTGAATTGCATTGAGAATATGATAGTGCCGCTTTTTA
TGTAGGGGAACTGCTTGCCGTCGCATGGATTTTTGATTATAGAGTATTTTAGTGTTGACTAACTAA
ACTCTTGGGGGTAAGCTATAGAGTAAAGTAACCGGCAATCAGCGGTAAAATAGCTATTTTTCAAGA
TTTTAAGATTGAATATTGGATTTTACTCTACACTCGAAACCATAGAGTTGAGTAGAATATTTGACG
AATGGGAAACACATCATCGAAATTTGGTGGTTCTACACTGTGCGGGATCGTCAGACACCGACTGTT
ACCTTTAAGTTGCATCCGTGAAAACAAGGGGCCAAGGCAGGACAGAGTAATCACTGGTCGGCGTTG
GAAGCCTGAAGAAGGCCCCGGACACGAACCTCTCGTGTCCCCACAGGTTGCTACCTCGGCCTTCTT
CCGCTGTGGGCTTAAGCGTTACCCCACGTAGCCGCTGCGCTGCCAAATGAAATGTCCTCATCACGA
TGACAGCGATGCAAACTACACAGACACACCAAGACCGAGGTGACCGCGAGTAGCTGACGCTACTCC
AGACCAATGACTCACAGGTACAGACTCAAACGACAATCCGGGTCCGATGCTGCCAGGCTTCGCCCC
GGATGTCGTTGTGAAGCATCGTCGCAGCGGACCAGCAGAGCCGGTCCTCAACACGGATGGAGCGGT
ATTATCAGTACGCTCCTATCTACAATGTGGACGCGAGGGGTCAAATTGGTTTCGGGACCCTCGCAT
TTGCCGGCTTCCACGCAGTCCGCACTTCTCCACGGACTGCATGCACACGCCTAATCAACAACAAGG
AATCCGGAAGATCGTTCCAGGAGGGACCCTCTCTACTGCTGGAATTACCATTACCGAGGTCACCCC
ACGGGTAACAGAATGGATACCTGACCTTCTTGAGGAACTACTGCCACGGAGTATTCAATCGGTTCG
AAAATTCATCCGGCAAGAAGACCCAGAAGTCCTCACGCACGCACGATACAACACCGTCTATCGCCG
TCTTCAAGAGGAAACCCTGAGGTTCGACCATCAAGAATGGTGCTCCACAACGGATATTTGGAGTGA
TGCAGAGGCTGAAGCGGTTGAATACGTAGAATCACTTGTCGAGTTCGCAGTCAAATATTCTGACGT
TGACGAAGATGATCTCGACGAACTTTCGGAGTATCACCAGCAGCGGTGCAAATCGCTTAAACAGAC
```

TCTCACTACGATCAGTACCGGACGTGGCCCACTCAACGCTGGCCTTGAAGCCCTCGCCAAGGACC
CGTACGACTCCACGATGAGCTCGATGACGCACCGCAACCGATCACGCTTGTCCTTGATGGCGAGTT
GTGGTCAAAACTCGACGATAGAGGAACAGGTATCCGAGCACTTGCAGCTATCGCCGTGCTCGGCTC
CACCTTCGACGTCCGCCTGGTTATTTCACCAGCGTTAGACGCCGCGATTGAGCGACGGTATCCAGA
CTGGTATGACTCCCATCTCCGTCTTACTGAAACCCGTGAGACCTCCTCTGTAGAATCAGCGGGTGG
CGACGGACAACCATCGGCGGAGCAGCTCGAGGAAGCATGGGAAGCAATACAGAACCTCCCAGAGGA
ATCAGGGAGACTCCGTCTTCTCCGGAACCTCCCTATAGAGGGCTCTCGAGACTATCGTGATCTCAA
GCAGGATGATGAGATTGACGTGCAGGCAGGGACTGTCGGCCGTTACATCCTCGATCTAGAAGAATT
AGGGCTTGTGGATATCGACCGTCGTGGACAATACAACAGCGCATCCCTCACCGGCTTAGGACAAGT
AGCAGTTGAGCAGTATGTCACCACGGACTACCGGGTGATCCATCCGACCCAATCGACGCTGGAGAC
GCATCTTACGCCGACCCCTCAGCCCCAAGCAAGTACAGTGTATCCCGCGCGATCGGACACGAGGGA
GGGGGATCAGCCTGGGACAGCGGAGGATTGGATAGCTGCGACAGGCAGTCCTAGTGAGGGTGCTGA
CTACGTTCAATGGCTCGATGGGCCGTCTGGTGTTCTCGACGCTTGGGGAATGCATCAGCGGTACCT
TGCTGGCCGTCGAGATCGTGGTGTCACCCTAGTCGATGACCGTATCGAGCGTTTCGAGGACGGTCG
CGTATCGTATCTCAGTTGCTTCGACGATGATCTCTTCGTAGCCACTCAGTGGGGCGGCCCCCTTCC
TACCCTGGGACGTATTGCGGGTGCCTTACTCAGCGACAAAGCACTGAGTAAGATCCTTACCCCTTC
TCGTCTCGGAAACCAGTTCGAAGAAATCAACGATGCGGTCGTTGAACAACTCGATAGAGAAGCTGG
GGAAATCATCCGTAGAGGACACCAAATCGGCTGGTTCAGCGAGGACGAAGAAGACTACGATGGTTG
GCGTGAGCGTATTGGCTCCGTTCGCAGTCTGTGTTTGCAACAAGTTGGTGAACTCACAAACAGTGA
CGACGTCGAAGCCCGGACTGAGTTACTACGTGACCTGCACGGCTTGGTTGCCTCAGCGACACAGCT
GTACTACGCAGCCGGTGTAGACGTCACAATCAACGTTCGAGTCCCTGACACGGGAATGCTCATCAG
CGATGAACGCCGTCTCGACGATTTCCTCGGTTTCGCCCGGTACAGCATTCCGAAACAGTCCGTGTA
CGGGATTCATTCGGGGTATAGGATGCTCCTCGAGGATCGACCGGAGAAACTCAAACGACGTCTTCC
CTACGAGGTGGATGATGCTGATTCCACAATGCATCTGACCGCATCGTGGGTTTTCTCCGGATCAAC
GATGATCGATCTTCACGATGACATCGAGGACGCCATTGAAATGGAGACCAACGAAATTCGTGAAGC
AATTGCTAATGGACAGGAGTCAGCACCGGTAATGGAAATCCCCGTCCAGATTGGGAACTCCTACTC
AGCAATTCGTAATCACGTCGAAGACTACGCTTCAGCGAAGAACTACCAGGTAGCTCACCAGGAGGA
TATTCACGAAGGAAAACAGGATCTCGAGCGACTCGTCCGCCTGTTCCTCCGTGTCCTCGGGACAGA
GGATCGACCACATCGAGCGTGTCCTCACGACGTTGCCGAGGCAATGCTTCATGTTGCTCAATCCTC
CCGGAACTACGATTTCATCACCGTGCGAGACATCTCGTACGGACTGTCGAATCTCCCCACGAAACG
ACTCTTACCCGAGCTCCCACCAACAGCAACGAAGCTCCTCAAAACCCTGCTTGATGCGGATGACCC
GATGGGACGGTCTGAAATCATCGATACCGCTGACATTTCGGAAAGTAGCTATGATCGCTACATCAA
CGAACTCGCCGCTTGGGATATCATTGAACCCCGCGAAATCGAGGGACACCGTCGGTGGGAAGCTCA
CTTAGAGCCGTGGTGGACACCGCAGAGTGATCGAGACGAACCCTATGCCGACCCTGACCCCGACAC
GGGAATACTGTACGCTGAATTTCCCCGTGATGTCGCTAGTGCGGTGATGTGCCACCTCATCACCCA
CTACGACTTACCCGACCTTGAGACAGCGTATCTTGAGGGTATCCAACCGGGGGACGACATCAAGGC
CCTCTTCGATGATCACGACCGACTCAGACGATGGCGGCCATTCCTCTGGGGTGCATTCGCCGACTC
GGACAAACTTGAAAGAGGCCCATCTGGTACAGCAGCTTCGGACTCGACCGTAGTTCGTCTCGGTCA
ATCTCCAGGACCCGACACAGCACAATCGAGCTTCCAAGACGTCTCAGAAACAGCAACCCAACGAGA
TCGTCTCAGTCAACCGTCGCCAGGGCTGGACTAATGATTGACGGCAATCGATAACTACGCAATGAA
TCCTCCACAACCAGCATCTGAACCGTACTCGAAAGGTGATGCAGTAACCGTATACGTGGGCGAAGA
CGACCCCGATGTCCGGTACCACGGCGTTAAATGCGTCGTCACTGACCGCCTGCAAGATGATTTGAA
CACCGAGACAGGCCGTGACCGTGACCAGCACCTCTACCGCATAAAAAGACGGTCAACGGGCAAGGT
TCAGTACCTCACAAAGCATTCTCGGCTAGCTGTTTCTGAAGCCTGAGTTCCACGGCGGAGCTGTTG
TGCTGGTGGTCTTGACGAGAAGATCTCCGACGAACCGACGGAGAGCTGTCGCTGTCGGCGGCGGCT
GCCGCCGACGCGACAGCGTCGCCACTCCTGGCGAAGGCTACCCTGGTCGGGGATTAGCGGGTAAAC
CGGTCGTCTGGCGGTCGGTTTGCGGGGACGGCCCGACGCGTCGCGAGGGCCGTCCACGCTGCCCGT
TGAATCATGTTGATGAACTCCTTGAACGACCACTTCCAGAGGCGACGCCCCCCACGGCGGGGCGTC
GCCACGTACTCCCAATGTAAATACCGCCAGACGTTTTGTAACAACAAGCTCACCACGACGTACAGC
AGCCGTACGACCGGATTTTGTGTTGAGGTCGTCGCAATACTTTGTTCAGAGAGTCGATAGCTCGCT
TCGATACCGAAGCGTTTCGCGTAGTGGTATCGAGCGTCGCGTGGACTCTCGATGAACGGCGCGTCA
GCGGCGTAGCCGTGACGCGCCACCCCATGTTCGTCGCACCGTCCGTTTTGGTACGTACAGTCGATG
TAGACGAGAAACTCGACGGTCCAGCTGTGACCGTCGAGTTTCGCCGTCATGTCGTGTTGAATGACG
CGACTCCAGCCTTCCGAGAGTTCTTGCTTGATCGTCTGTCCCCAGCGGACGATCGGCATGACGTAG
GCGTGGTTGTGTGCCTGAAGCAGCGTCAAACACTTGCTGTCGTAGAATTGGGATCTCCCGCTGGCC
CTTCGCCAGCTCCTCTGCGATCCCCGGCTCGTCACCGAGTGTCGACTGGAACGAGGTCATTTCCTG
ATGCGCATCTACCCAGTCCCGCGTTAAGTACTCACCGGCAGCGGAGTGAAAGTGAACTCGGGGCGT
TGACGCGCTTCGAGGGGCGTTCGCGCCGGAACGCCGAATCATGATACACAGAGTCACCCACGAAAC
GCGGGTCGGTCGGTCGGCGTCTCCGATGTCGGAAACCGCTACGCGGACTCCAGTAAGTAGTACGC
GCCCGCGCGTGCGAGCCTTTAAGGGACGGGCTATCCTAGGCCTGTTCAGATGTCTCAGGATAACGA

GTACGGGGCCGGACAGATTCAGGTCCTCGAAGGGCTTGAAGCCGTTCGAAAGCGTCCGGCGATGTA
CATCGGGTCCACCGATTCTCGCGGACTCCACCACCTCGTCTACGAAGTCGTCGACAACTCCATCGA
CGAGGCGCTCGCGGGCCACTGCGACGCCATCGAGGTCGCCCTCCACGAGGACGGCTCTGTCAGCGT
CACCGACAACGGCCGCGGGATTCCGGTAGGTACGCACGAACAGTACGACCGACCCGCGCTGGAGGT
CATCATGACCGTCCTCCACGCCGGCGGGAAGTTCGACAACAAGTCCTACCAAGTCTCCGGCGGCCT
CCACGGCGTCGGCGTCACCGTCGTCAACGCGCTGTCGAGCGAACTCGAAGTCGAGGTCAAACACGA
CGGCGCGGTGTGGACACACCGCTTCGAGGTCGGCGAGCCGCAGGTCGAGGAGTTCGAGCGCGTGCG
CGACCTCGAACCCGGCGAAGACACCGGAACGACCATCCGCTTTTGGCCGGACGACGGTATCTTCGA
GACGACCGAGTTCGACTTCAAGACGCTCGAAAACCGCCTGCGAGAGCTCGCGTTCCTCAACTCCGG
GGTCGAGATTTCGCTCTCCGACGAGCGGACCGACGAGTCGAGCACGTTCCTCTTCGAGGGCGGCAT
CCGCGAGTTCGTCGAGTACCTCAACGAGACCAAGACGGCGCTCCACGACGACGTCATCTACTACGA
CGACGAGTCGGAGGGCATCGAGGTCGAAATCGCCATGCAGGCGACCGACGAGCTCCAGGGGTCGAT
TCACGCCTTCGCCAACAACATCAACACCCGCGAGGGCGGCACGCATCTGACCGGGTTCAAGACGGC
GCTCACCCGCGTCGTCAACGACTACGCGAACAGCCACGACATGCTGGACGACCTCGACGGCGACAA
CCTCCGCGGCGAGGACGTTCGCGAGGGACTCACCGCCGTCATCCCCGTCAAGCACCCCGACCCGCA
GTTCGAGGGCCAGACGAAGACCAAGCTCGGCAATTCCGAGGTCCGCGGCATCGTCGAGAGCGTCAC
CCACCAACAGCTCGGGACGTTCTTCGAGGAGAACCCGGACACGGCGACGGCCATCATCTCGAAGGC
CGTCGAGGCCGCCCGCGCCCGCAAGGCCGCGAAGCAGGCCGAGGAGCTCACCCGCCGCAAGTCCGC
GCTCGAATCTACCTCGCTGCCGGGGAAGCTCGCGGACTGCCAGAGCCGCGACCCCTCGGAGTCCGA
ACTGTTCATCGTGGAGGGCGACTCCGCGGGCGGGTCGGCCAAGCAGGGCCGCGACCGCAAGTTCCA
GGCGATTTTGCCCCTCAAGGGGAAGATTCTGAACGTCGAGAAACACCGCCTCGACCGCATTCTCGA
AAACGACGAGATACGGGCGCTCATCACCGCCATTGGCGGCGGCGTCGGCGACGAGTTCGACATCGA
GAAGGCGCGCTACCAGCGGCTCATCCTGATGACCGACGCCGACGTCGACGGCGCGCACATCCGGAC
GCTGCTGCTTACGCTTCTGTACCGGCACATGCGCCCGCTCATCGAGGCCGGCTACGTGTACGCGGC
TCAACCGCCGCTGTACCGCGTCCGCTACCGCGGCAACACCTACGACGCGATGGACGAGGCCGAGCG
GGACCGCATCATCGAGGAGGAATGCAACGGCAACCCCACGCAGGTCCAGCGGTTCAAGGGACTCGG
TGAGATGAACCCCGACCAGCTGTGGGACACGACGATGAACCCCGAAAACCGCGTCCTCAAGCGCAT
CACCGTCGAGGACGCCGCCGCCGCCGACCGGATGTTCAACATCCTGATGGGCGACGCCGTCGGGCC
GCGAAAGCAGTTCATCAAGGACCACGCGAACGACGCCGAATGGGTAGACATCTAATATGAGTTCTG
ACGCACCAGATTCGTTCGAACCGCCCAAAAGCGCCCAATACGCAAACCGCCTCTCCCCGCGCGTTG
GCCGATTCATTAATGCAGCTGGCACGACAGGTTTCCCGACTGGAAAGCGGGCAGTGAGCGCAACGC
AATTAATGTGAGTTAGCTCACTCATTAGGCACCCCAGGCTTTACACTTTATGCTTCCGGCTCGTAT
GTTGTGTGGAATTGTGAGCGGATAACAATTTCACACAGGAAACAGCTATGACCATGATTACGCCAA
GCTTGGTACCGAGCTCGGATCCACTAGTAACGTTAATTAAGCCCGGGCCGGCCGTTAACCTGCAGG
ATTGCAGATATCCATCACACTGGCGGCCGCTCGAGCATGCATCTAGAGGGCCCAATTCGCCCTATA
GTGAGTCGTATTACAATTCACTGGCCGTCGTTTTACAACGTCGTGACTGGGAAAACCCTGGCGTTA
CCCAACTTAATCGCCTTGCAGCACATCCCCCTTTCGCCAGCTGGCGTAATAGCGAAGAGGCCCGCA
CCGATCGCCCTTCCCAACAGTTGCGCAGCCTGAATGGCGAATGGACGCGCCCTGTAGCGGCGCAT
TAAGTTTGGGCAATTCGTGGACTCTTGTTCCAAACTGGAACAACACTCAACCCTATCGCGGTCTAT
TCTTTTGATTTATAAGGGATTTTGCCGATTTCGGCCTATTGGTTAAAAAATGAGCTGATTTAACAA
ATTCAGGGCGCAAGGGCTGCTAAAGGAACCGGAACACGTAGAAAGCCAGTCCGCAGAAACGGTGCT
GACCCCGGATGAATGTCAGCTACTGGGCTATCTGGACAAGGGAAAACGCAAGCGCAAAGAGAAAGC
AGGTAGCTTGCAGTGGGCTTACATGGCGATAGCTAGACTGGGCGGTTTTATGGACAGCAAGCGAAC
CGGAATTGCCAGCTGGGGCGCCCTCTGGTAAGGTTGGGAAGCCCTGCAAAGTAAACTGGATGGCTT
TCTTGCCGCCAAGGATCTGATGGCGCAGGGGATCAAGATCTGATCAAGAGACAGGATGAGGATCGT
TTCGCATGATTGAACAAGATGGATTGCACGCAGGTTCTCCGGCCGCTTGGGTGGAGAGGCTATTCG
GCTATGACTGGGCACAACAGACAATCGGCTGCTCTGATGCCGCCGTGTTCCGGCTGTCAGCGCAGG
GGCGCCCGGTTCTTTTTGTCAAGACCGACCTGTCCGGTGCCCTGAATGAACTGCAGGACGAGGCAG
CGCGGCTATCGTGGCTGGCCACGACGGGCGTTCCTTGCGCAGCTGTGCTCGACGTTGTCACTGAAG
CGGGAAGGGACTGGCTGCTATTGGGCGAAGTGCCGGGGCAGGATCTCCTGTCATCTCGCCTTGCTC
CTGCCGAGAAAGTATCCATCATGGCTGATGCAATGCGGCGGCTGCATACGCTTGATCCGGCTACCT
GCCCATTCGACCACCAAGCGAAACATCGCATCGAGCGAGCACGTACTCGGATGGAAGCCGGTCTTG
TCGATCAGGATGATCTGGACGAAGAGCATCAGGGGCTCGCGCCAGCCGAACTGTTCGCCAGGCTCA
AGGCGCGCATGCCCGACGGCGAGGATCTCGTCGTGATCCATGGCGATGCCTGCTTGCCGAATATCA
TGGTGGAAAATGGCCGCTTTTCTGGATTCAACGACTGTGGCCGGCTGGGTGTGGCGGACCGCTATC
AGGACATAGCGTTGGATACCCGTGATATTGCTGAAGAGCTTGGCGGCGAATGGGCTGACCGCTTCC
TCGTGCTTTACGGTATCGCCGCTCCCGATTCGCAGCGCATCGCCTTCTATCGCCTTCTTGACGAGT
TCTTCTGAATTGAAAAAGGAAGAGTATGAGTATTCAACATTTCCGTGTCGCCCTTATTCCGCAGGA
AAGAACATGTGAGCAAAAGGCCAGCAAAAGGCCAGGAACCGTAAAAAGGCCGCGTTGCTGGCGTTT
TTCCATAGGCTCCGCCCCCCTGACGAGCATCACAAAAATCGACGCTCAAGTCAGAGGTGGCGAAAC

CCGACAGGACTATAAAGATACCAGGCGTTTCCCCCTGGAAGCTCCCTCGTGCGCTCTCCTGTTCCG
ACCCTGCCGCTTACCGGATACCTGTCCGCCTTTCTCCCTTCGGGAAGCGTGGCGCTTTCTCAATGC
TCACGCTGTAGGTATCTCAGTTCGGTGTAGGTCGTTCGCTCCAAGCTGGGCTGTGTGCACGAACCC
CCCGTTCAGCCCGACCGCTGCGCCTTATCCGGTAACTATCGTCTTGAGTCCAACCCGGTAAGACAC
GACTTATCGCCACTGGCAGCAGCCACTGGTAACAGGATTAGCAGAGCGAGGTATGTAGGCGGTGCT
ACAGAGTTCTTGAAGTGGTGGCCTAACTACGGCTACACTAGAAGGACAGTATTTGGTATCTGCGCT
CTGCTGAAGCCAGTTACCTTCGGAAAAAGAGTTGGTAGCTCTTGATCCGGCAAACAAACCACCGCT
GGTAGCGGTGGTTTTTTTGTTTGCAAGCAGCAGATTACGCGCAGAAAAAAAGGATCTCAAGAAGAT
CCTTTGATCTTTTCTACGGGGTCTGACGCTCAGTGGAA

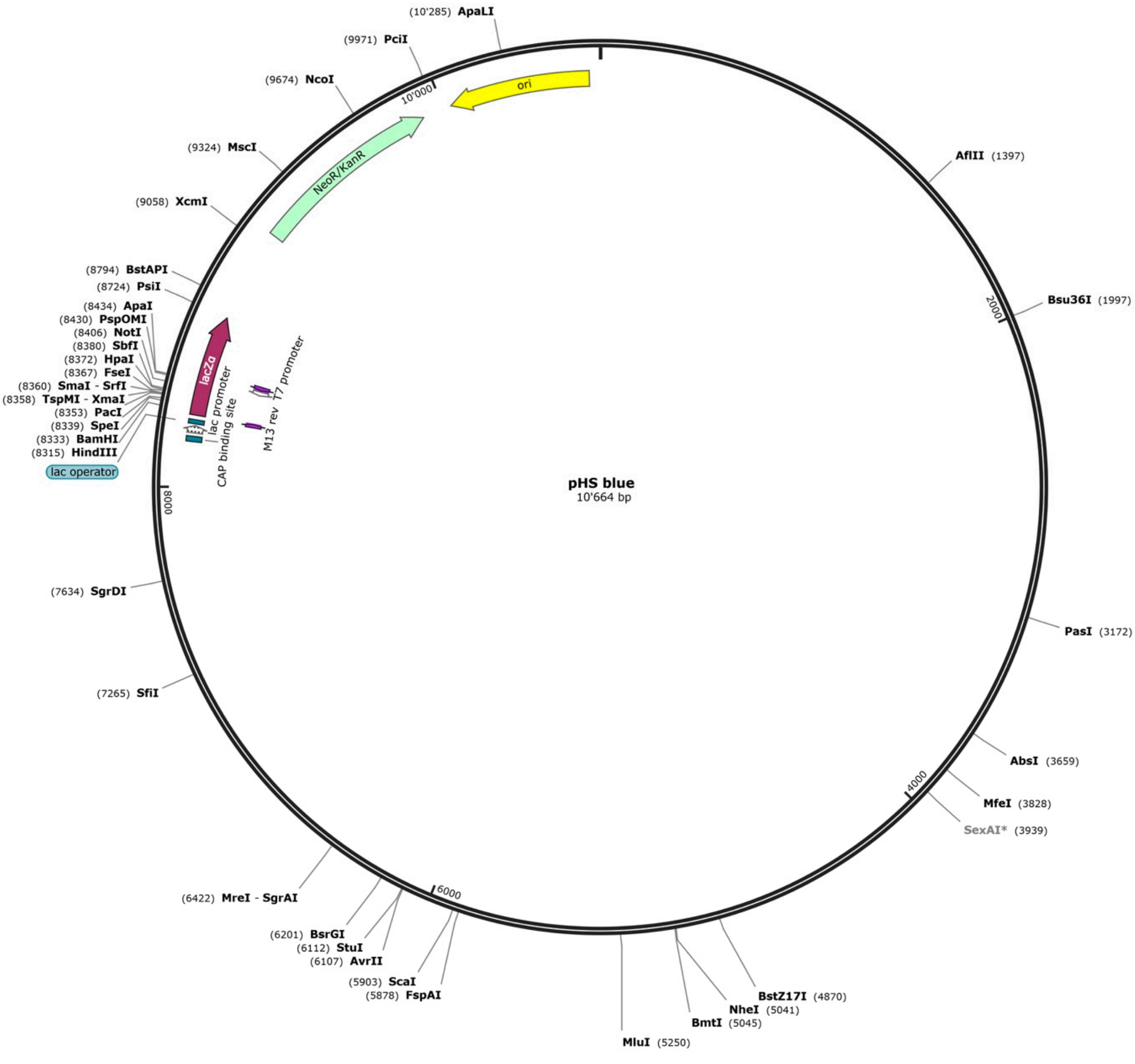

Figure S1: Vector map of pHS blue
